# Metal Emulsion-Based Synthesis, Characterization, and Properties of Sn-Based Microsphere Phase Change Materials

**DOI:** 10.3390/molecules26247449

**Published:** 2021-12-09

**Authors:** Xiali Zheng, Wei Luo, Yun Yu, Zebin Xue, Yifan Zheng, Zongjian Liu

**Affiliations:** College of Chemical Engineering, Zhejiang University of Technology, Hangzhou 300014, China; 2111901021@zjut.edu.cn (X.Z.); 2111801030@zjut.edu.cn (W.L.); 2111901022@zjut.edu.cn (Y.Y.); 2112101003@zjut.edu.cn (Z.X.); zhengyifan@zjut.edu.cn (Y.Z.)

**Keywords:** molten salts, metal emulsion, microspheres, interfacial reaction, ultrasound

## Abstract

A comparative study of the metal emulsion-based synthesis of Sn-based materials in two different types of molten salts (namely LiCl–KCl–CsCl and LiNO_3_-NaNO_3_-KNO_3_ eutectics) is presented, and the properties of Sn, Sn-Cu and Sn-Cu-Zn microsphere phase change materials prepared in chloride salts are evaluated by differential scanning calorimetry (DSC) to understand the effect of element doping. Despite a high ultrasonic power (e.g., 600 W or above) being required for dispersing liquid Sn in the chloride system, well-shaped Sn microspheres with a relatively narrow size range, e.g., about 1 to 15 µm or several micrometers to around 30 µm, can be prepared by adjusting the ultrasonic power (840–1080 W), sonication time (5–10 min) and the volume ratio of salts to metal (25:1–200:1). Such a method can be extended to the synthesis of Sn-based alloy microspheres, e.g., Sn-Cu and Sn-Cu-Zn microspheres. In the nitrate system, however, a very low ultrasonic power (e.g., 12 W) can be used to disperse liquid Sn, and the particles obtained are much smaller. At low ultrasonic power (e.g., 12 W), the particle size is generally less than 10 or 4 µm when the sonication time reaches 2 or 5 min, and at high ultrasonic power, it is typically in the range of hundreds of nanometers to 2 µm, regardless of the change in ultrasonic power (480–1080 W), irradiation time (5–10 min), or volume ratio of salts to metal (25:1–1000:1). In addition, the appearance of a SnO phase in the products prepared under different conditions hints at the occurrence of a reaction between Sn droplets and O_2_ in situ generated by the ultrasound-induced decomposition of nitrates, and such an interfacial reaction is believed to be responsible for these differences observed in two different molten salt systems. A DSC study of Sn, Sn-Cu, and Sn-Cu-Zn microspheres encapsulated in SiO_2_ reveals that Cu (0.3–0.9 wt.%) or Cu-Zn (0.9 wt.% Cu and 0.6% Zn) doping can raise the onset freezing temperature and thus suppress the undercooling of Sn, but a broad freezing peak observed in these doped microspheres, along with a still much higher undercooling compared to those of reported Sn-Cu or Sn-Cu-Zn solders, suggests the existence of a size effect, and that a low temperature is still needed for totally releasing latent heat. Since the chloride salts can be recycled by means of the evaporation of water and are stable at high temperature, our results indicate that the LiCl–KCl–CsCl salt-based metal emulsion method might also serve as an environmentally friendly method for the synthesis of other metals and their alloy microspheres.

## 1. Introduction

The mismatch between energy supply and demand is one of main handicaps to the widespread use of renewable energy. Thermal energy storage (TES) provides an efficient means for solving this problem, and is a key technology for the efficient utilization of energy [1]. Generally, TES can be classified into three classes, namely sensible heat storage, latent heat storage and thermochemical energy storage, and the former two are much more fully developed than the latter. In comparison with sensible heat storage, latent heat storage has the advantages of high storage density and the quasi-isothermal nature of the storage process. In most latent heat storage systems, heat storage/release is achieved via the cyclic solid–liquid phase transition of so-called phase change materials (PCMs). Depending on the working temperature, a wide range of materials can be used as solid–liquid PCMs, e.g., organics [2], inorganic salts [3] and metals [4]. Among them, the organic PCMs generally have a low melting point and thus are commonly used in low-temperature TES (<220 °C). In contrast, most inorganic salts and metals possess a high melting point and are used in medium-(220–420 °C) or high-temperature TES (>420 °C). Although many medium–high-temperature TES systems are based on molten inorganic salts, they suffer due to the low thermal conductivity of the PCMs, and thus the overall heat transfer rate is relatively poor. This problem can be overcome by using metal-based PCMs, because their thermal conductivities are much higher than those of inorganic salts [4]. Even for the metal Bi with a very poor thermal conductivity, for example, its thermal conductivity (about 8 W⋅m^−1^⋅K^−1^) is much higher than that of the available commercial molten salts (less than 0.6 W⋅m^−1^⋅K^−1^) [3]. As a result, metal-based PCMs have recently drawn increasing attention [5,6,7,8,9,10].

Metal Sn has a melting point of about 231 °C and a density of about 7.3 g/cm^3^. Although its latent heat of fusion per unit mass (closed to 60 kJ/kg) is lower than that of inorganic salts [3], its high density provides it with a storage density on a volume basis that is comparable to those of many inorganic salts. In particular, its much higher thermal conductivity (about 67 W⋅m^−1^⋅K^−1^) gives Sn an edge as a PCM compared to inorganic salts. Furthermore, the melting point of Sn can be adjusted by means of the addition of a suitable amount of another metal element, such as Zn or Bi. Based on the phase diagram of Sn-Zn [11] or Sn-Bi [12], for example, the Sn-Zn eutectic and Sn-Bi eutectic have a melting point of about 200 and 140 °C, respectively. Therefore, Sn-based nanoparticles or microparticles should be potential PCMs for low- or medium-temperature latent heat storage and have drawn some attention recently [8,10]. Zhu et al. investigated Sn particles embedded in Al_2_O_3_ and found that the thermally stable Sn@Al_2_O_3_ particles are suitable for rapid thermal energy storage [8]. More recently, Bao et al. reported a study on using micro-sized Sn encapsulated in SiO_2_ as PCMs [10]. To use Sn microparticles, especially Sn-based multicomponent metal microparticles, as PCMs for latent heat storage, the first issue that needs to be addressed is how to prepare these microparticles with well-shaped morphology. Although Friedman et al. reported that well-shaped Sn microspheres could be prepared by ultrasonically dispersing liquid Sn into silicone oil [13], the use of silicone oil as a dispersion medium has several drawbacks, including that it tends to evaporate and even decompose at high temperatures and that harmful organics are needed for its removal from the surface of metal particles due to its very high viscosity. Therefore, the search for a simple and environmentally friendly method for the synthesis of well-shaped Sn-based microparticles is of great importance.

In this article, an ultrasound-assisted molten salts-based metal emulsion method was employed to prepare Sn-based microsphere PCMs, and the phase change properties of as-prepared microspheres were evaluated to investigate the effect of element doping. A metal emulsion is a special dispersal system in which liquid metals serve as the dispersed phase. A typical example is steel droplets dispersed into the slag observed in the steelmaking process. This special emulsion plays an important role in controlling the rate of reaction between molten steel and slag, and in order to clarify its formation mechanism researchers have studied the bubbling-assisted emulsification of liquid metal with molten chloride salts as the slag phase [14,15,16]. These studies demonstrated that micro-sized emulsified metal liquid droplets can be extracted by cooling them and then dissolving the salts into water. Unlike the traditional oil–water emulsions, however, this molten salts-based metal emulsion is highly unstable because of the high interfacial tension between liquid metal and molten salts [17,18,19,20,21], the ionic nature of the molten salt, the high emulsification temperature at which no surfactant can be added, as well as the much lower viscosity of molten salts compared to silicone oil used in the work by Friedman et al. [13]. Furthermore, the high density of Sn-based metals causes a large density difference between liquid metal (about 7.0 g/cm^3^) and molten salts (about 2.0 g/cm^3^), which may further increase the instability of the emulsion. Hence, our understanding of this molten salt-based metal emulsion method is very limited, and some questions remain when we adopt this molten salt-based metal emulsion method to prepare Sn-based microspheres. For example, can we use this method to prepare Sn-based microspheres with a small size and a narrow size distribution despite high instability of this metal emulsion? What will occur when the chloride salts are replaced by nitrate salts, in which a reaction might occur at the interface between liquid metal and molten nitrate salts caused by acoustic cavitation? Therefore, the content of the present work mainly includes three aspects. First, the operating parameters, such as the ultrasonic power, sonication time, and volume ratio of salts to metal, were varied to show the possibility of controlled synthesis of Sn-based microspheres with a small size and a narrow size distribution. Second, although LiNO_3_-NaNO_3_-KNO_3_ eutectic was reported to be stable at a temperature of 425 °C [22,23], a value above our experimental temperature, the instantaneous local high temperature caused by acoustic cavitation might induce the occurrence of decomposition of nitrates, and thus the effect of the replacement of LiCl–KCl–CsCl eutectic with LiNO_3_-NaNO_3_-KNO_3_ eutectic on the size, morphology, phase composition and microstructure of the product was evaluated. Third, by encapsulating the as-prepared microspheres in SiO_2_, the phase change properties of Sn-based microspheres were investigated.

## 2. Results and Discussion

### 2.1. In LiCl–KCl–CsCl Eutectic System

LiCl–KCl–CsCl eutectic has a composition of 54.4 wt.% CsCl, 30.3 wt.% LiCl, and 15.3 wt.% KCl. Since this ternary eutectic is completely molten at a temperature close to 280 °C, an initial temperature of about 300 °C (for pure Sn) or above (for Sn-based alloys) is chosen to emulsify the liquid metal into molten chloride salts. In this section, we first study the effect of the preparation parameters, such as ultrasonic power, sonication time, and the volume ratio of salts to metal, on the size and morphology of resulting metal Sn particles. Then, the result obtained is extended to the synthesis of Sn-based alloy microspheres.

#### 2.1.1. The Effect of Ultrasonic Power, Sonication Time, and the Volume Ratio of Salts to Metal

**Figure 1:**
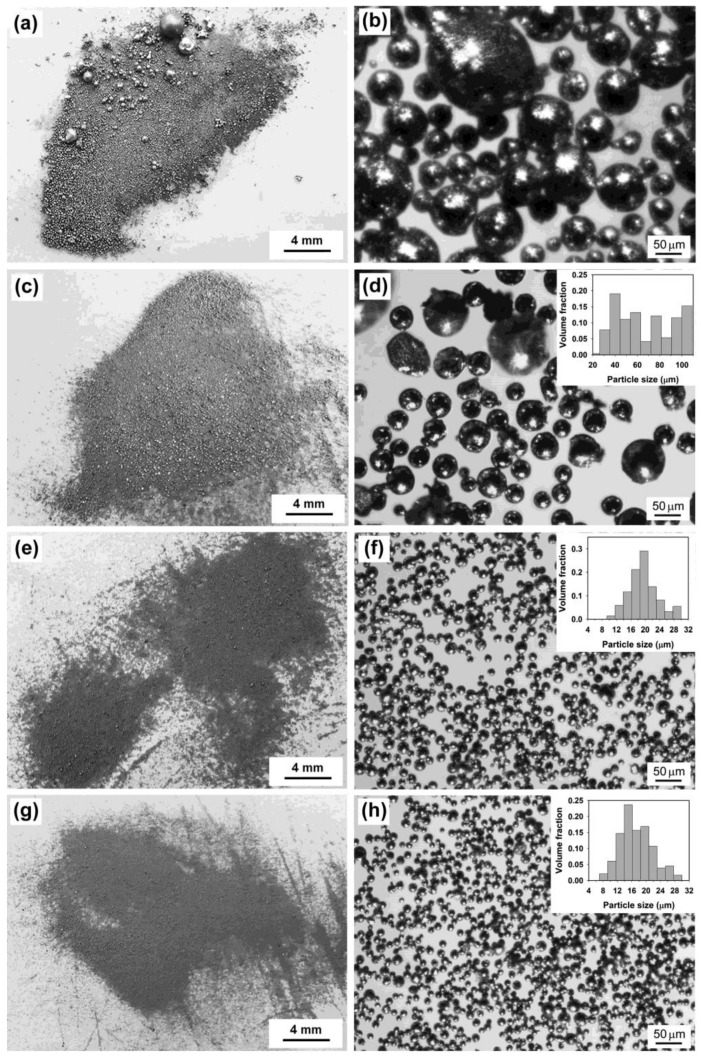
Optical images of the products obtained under different ultrasonic powers: (**a**,**b**) 300 W, (**c**,**d**) 600 W, (**e**,**f**) 840 W, and (**g**,**h**) 1080 W. Note that (**b**) is the image of small Sn particles present in (**a**). Other experimental conditions: volume ratio of salts to metal = 25:1 and sonication time = 5 min. Inset is the corresponding volume-based particle size distribution.

**Figure 2:**
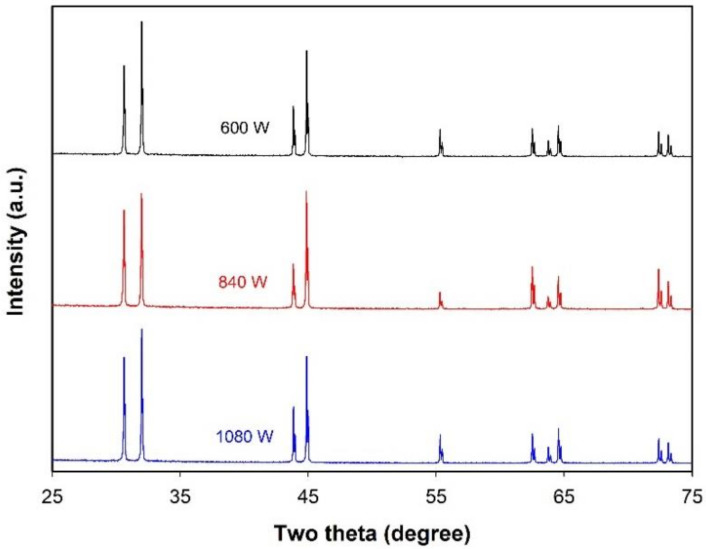
XRD patterns of the products obtained under different ultrasonic powers. Other experimental conditions: volume ratio of salts to metal = 25:1 and sonication time = 5 min.

Figure 1 presents the optical images of the products formed under different ultrasonic powers. At a power of 300 W, the liquid Sn cannot be completely dispersed into the molten salts by ultrasonic waves, and thus bulk Sn with a size of several millimeters is present in the product (Figure 1a), despite the fact that the size of small particles is in the range of tens of micrometers to hundreds of micrometers (Figure 1b). When the ultrasonic power is raised to 600 W, however, this phenomenon disappears (Figure 1c) and spherical particles with sizes typically ranging from about 20 µm to more than 100 µm (mainly in the range of 25–65 µm) can be observed (Figure 1d and its inset). The fact that a high acoustic power is needed for completely dispersing liquid Sn into molten chloride salts could be related to the high interfacial tension between liquid metal and molten salts. For instance, the interfacial tension between liquid Al and molten NaCl-KCl eutectic is about 750 mN/m at 800 °C [17], much higher than those between oil and water, e.g., the interfacial tension between cyclohexane and water is 50.38 mN/m at 25 °C [24]. Since the energy needed for expanding the metal–salt interface is directly proportional to the value of interfacial tension, a high interfacial tension requires a high ultrasonic power for preparing an emulsion. As shown in Figure 1e,f, further increasing the ultrasonic power to 840 W can lead to the formation of microspheres of a much smaller size: particles of 12–25 µm in diameter dominate in the product. This result can be explained by the fact that a higher power delivered to an ultrasonic transducer can produce a stronger amplitude of ultrasonic waves which can increase the rate at which emulsion droplets are broken up into smaller droplets. However, when the power was increased to 1080 W, only a slight reduction in the size of the formed microspheres (the dominant particle size range is 10–24 µm) can be observed (Figure 1g,h). It is well-known that the droplets formed in the emulsion are controlled by the interplay between droplet breakup and droplet coalescence. No significant change in the size of the formed Sn microspheres suggests that, although a high ultrasonic power generally favors the droplet breakup, the coalescence of Sn droplets in the emulsion also becomes evident when the size of droplets is small. The XRD patterns of the products formed at different ultrasonic powers are presented in Figure 2. All the products exhibit similar diffraction patterns, and the peaks observed at 2θ of 30.6°, 32.0°, 43.9°, 44.9°, 55.4°, 62.5°, 63.8°, 64.6°, 72.4° and 73.1° can all be indexed to metal Sn with a tetragonal structure (space group I41/amd, JCPDS 04-0673) and correspond to the diffractions of the (200), (101), (220), (211), (301), (112), (400), (321), (420) and (411) planes, respectively. The absence of the diffraction peaks of SnO or SnO_2_ indicates that the microspheres formed are Sn microspheres and that the Ar gas protection can avoid the oxidation of Sn droplets during ultrasonic emulsification.

**Figure 3:**
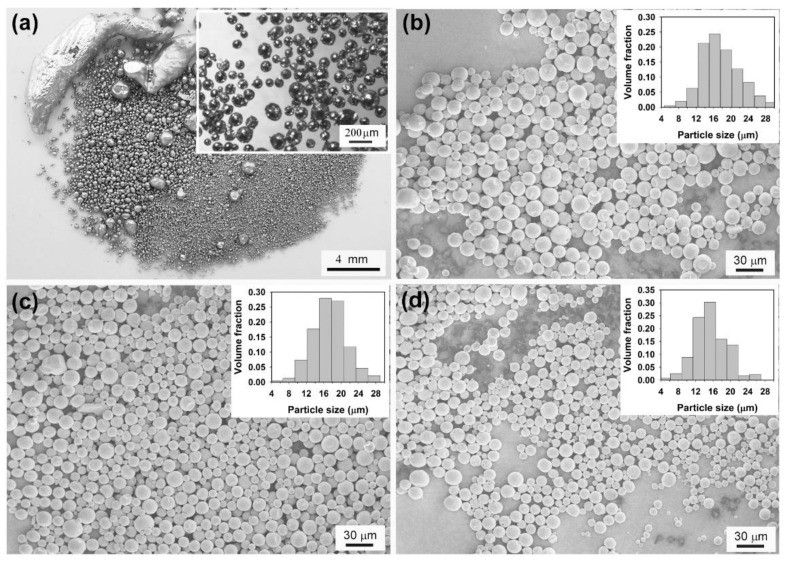
Optical or SEM images of the products obtained under different volume ratios of salts to metal: (**a**) 5:1, (**b**) 25:1, (**c**) 100:1, and (**d**) 200:1. Other experimental conditions: ultrasonic power = 1080 W and sonication time = 5 min. Inset is the optical image of Sn particles of small size present in the product or the volume-based particle size distribution.

In order to obtain droplets of small size in the emulsion, the factors which can prevent droplet coalescence should be considered. In traditional emulsions, the coalescence of droplets can be controlled by the addition of surfactant. In metal emulsions, however, no surfactant can be used in such an ionic, high-temperature system. Since a high volume ratio of salts to metal can increase the distance between Sn droplets, it is expected that the rise in the volume ratio of salts to metal is unfavorable for the coalescence of droplets. To study the possibility of the synthesis of Sn metals of smaller size, therefore, the volume ratio of salts to metal was raised from 25:1 to 200:1. Figure 3 presents the optical or SEM images of the products formed under different volume ratios of salts to metal. As can be seen from Figure 3a, a decrease in the volume ratio of salts to metal from 25:1 to 5:1 can give rise to bulk Sn being present in the product, indicating that droplet coalescence dominates in this case. However, the upper size range of Sn microparticles is only slightly changed from about 30 µm to around 26 µm when the volume ratio of salts to metal is raised from 25:1 to 200:1 (see Figure 3b–d), suggesting that under our experimental conditions, a volume ratio of salts to metal of 25:1 is high enough to prevent the coalescence of most droplets in the emulsion, and thus raising the volume ratio of salts to metal is not an effective way to reduce the size of Sn microspheres (despite the fact that the dominant particle size range shifts very slightly to the left) when its value reaches 25:1.

**Figure 4:**
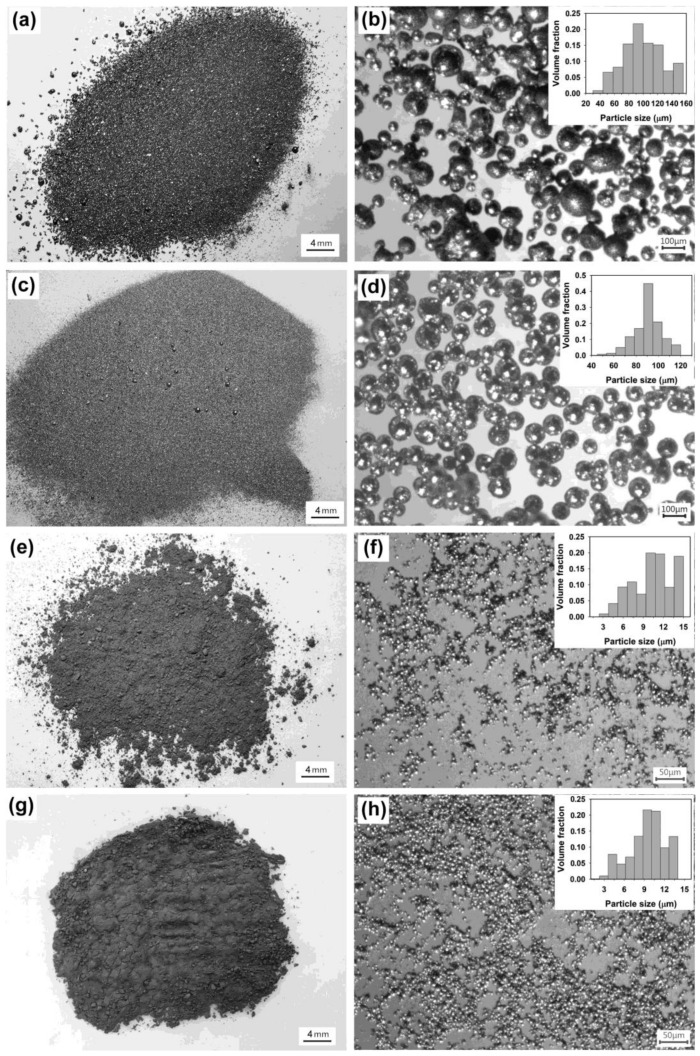
Optical images of the products obtained under different irradiation times: (**a**,**b**) 1 min, (**c**,**d**) 2 min, (**e**,**f**) 10 min, and (**g**,**h**) 15 min. Other experimental conditions: ultrasonic power = 1080 W and volume ratio of salts to metal = 25:1. Inset is the corresponding volume-based particle size distribution.

**Figure 5:**
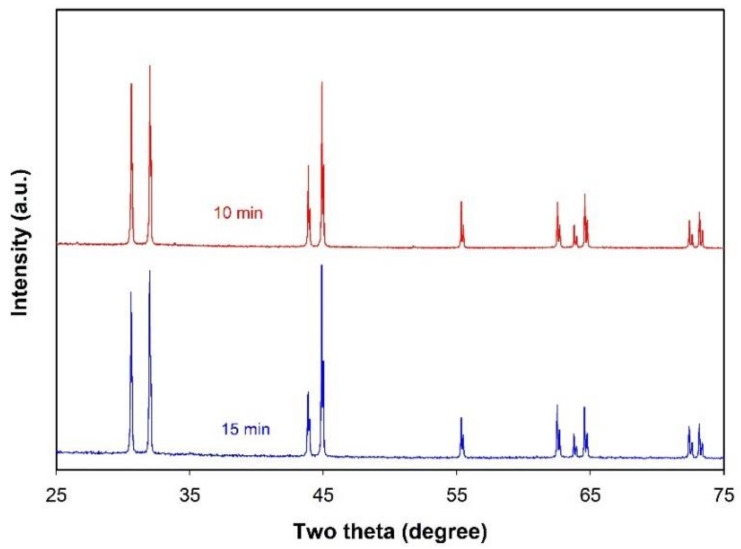
XRD patterns of the products obtained under different sonication times. Other experimental conditions: ultrasonic power = 1080 W and volume ratio of molten salts to liquid Sn = 25:1.

To further adjust the size of Sn microspheres, the effect of sonication time was also investigated at constant ultrasonic power. Although prolonging the sonication time might also favor the coalescence of metal droplets, the breakup of big droplets into small ones is strongly related to the sonication time when the applied ultrasonic power is fixed. Figure 4 presents the optical images of the products formed under different sonication times. It is obvious that the sonication time plays an important role in determining the particle size. At a short sonication time, e.g., 1 or 2 min, the size of Sn particles is typically in the range of tens of micrometers to more than 150 μm (Figure 4a,b) or about 10 to 100 µm (Figure 4c,d). When the sonication time is raised to 10 min, the size of the product is greatly reduced (Figure 4e,f), typically in the range of 1–15 µm. However, after a further increase in sonication time, e.g., 15 min, no obvious change in the size of the microspheres can be observed. The XRD patterns of the products formed at sonication times of 10 and 15 min are illustrated in Figure 5. Similarly to the XRD patterns of the product obtained at a sonication time of 5 min (blue curve in Figure 2), all the peaks observed can all be indexed to metal Sn with a tetragonal structure (space group I41/amd, JCPDS 04-0673), suggesting that these small-sized microspheres are still Sn microspheres.

#### 2.1.2. Preparation of Sn-Based Alloy Microspheres

**Figure 6:**
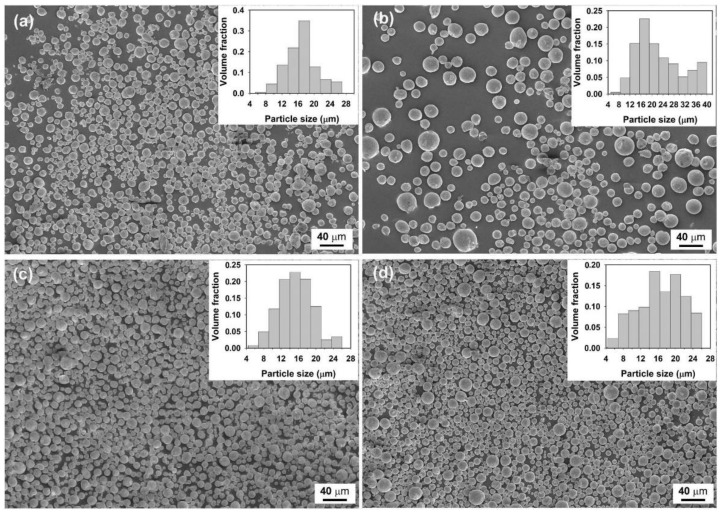
SEM images of the as-prepared Sn-based alloy microspheres of different chemical composition: (**a**) Sn-0.3Cu, (**b**) Sn-0.6Cu, (**c**) Sn-0.9Cu, and (**d**) Sn-0.9Cu-0.6Zn. Experimental conditions: ultrasonic power = 960 W, volume ratio of molten salts to liquid Sn = 25:1, and sonication time = 5 min. Inset is the corresponding volume-based particle size distribution.

**Figure 7:**
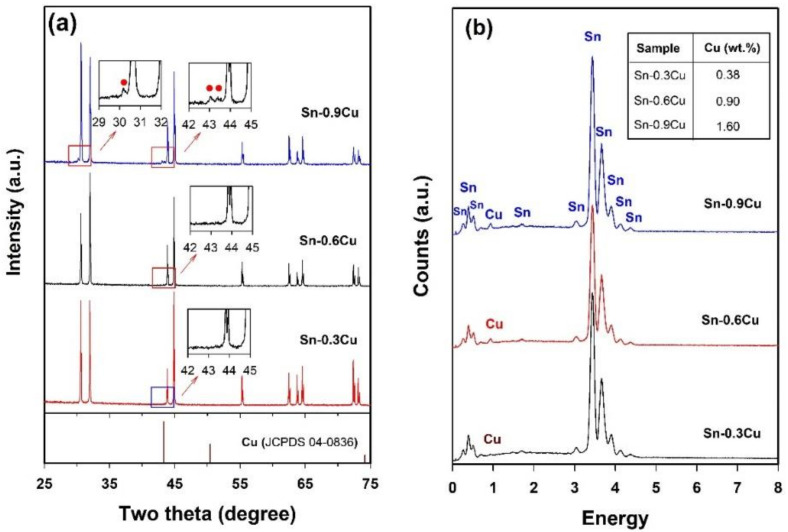
XRD patterns (**a**) and EDS spectra (**b**) of the Sn-Cu microspheres of different chemical compositions. The XRD data of Cu phase (JCPDS 04-0836) from the standard file is also shown for comparison.

Our above investigations suggest that well-shaped Sn microspheres can be prepared in the LiCl–KCl–CsCl system and Sn microspheres with a small size and a narrow size distribution can be obtained by adjusting the preparation parameters. We now extend this method to produce Sn-Cu and Sn-Cu-Zn alloy microspheres. Figure 6 shows the morphology of as-prepared Sn-based alloys of different chemical compositions, namely Sn-0.3Cu, Sn-0.6Cu, Sn-0.9Cu, and Sn-0.9Cu-0.6Zn (in wt.%, unless specified otherwise)_._ It is clear from Figure 6 that all the products are composed of microspheres with diameters typically in the range of several to about 27 µm, except for the Sn-0.6Cu sample, which contains some microspheres with diameters ranging from 30 to 40 µm. Since these microspheres were prepared under the same conditions, the bigger particle size observed in Sn-0.6Cu sample indicates that the chemical compositions might also affect the droplet size in the emulsion. As indicated by red solid cycles in the insets of Figure 7a, an intermetallic phase Cu_6_Sn_5_ can be detected by XRD in the Sn-0.9Cu microspheres. Although no Cu phase or Sn-Cu intermetallic phase can be found in Sn-0.3Cu and Sn-0.6Cu microspheres (Figure 7a), their EDS spectra confirm the existence of Cu on the surface of the microspheres (Figure 7b). In addition, as shown in the inset of Figure 7b, the surface Cu concentrations of these Sn-Cu microspheres obtained from their EDS spectra are generally higher than the nominal concentrations of Cu, hinting that Cu tends to preferentially segregate onto the surface of the microspheres.

**Figure 8:**
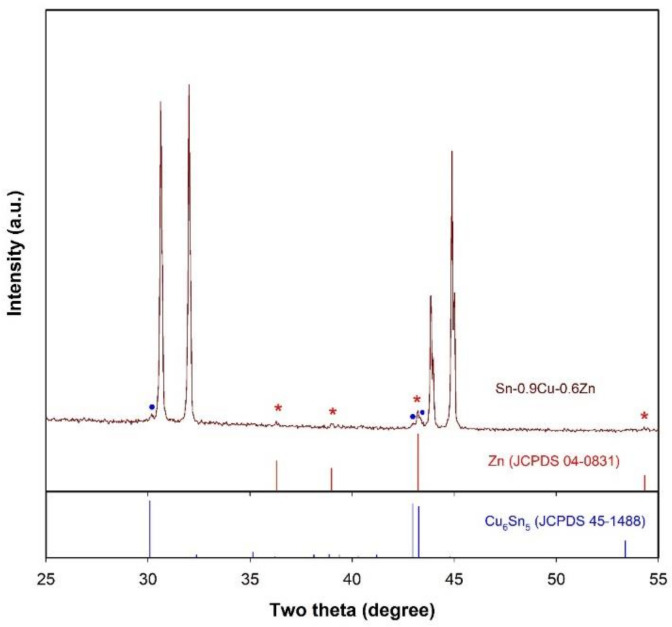
XRD patterns of Sn-0.9Cu-0.6Zn microspheres. The XRD data of Zn phase (JCPDS 04-0831) and Cu_6_Sn_5_ phase (JCPDS 45-1488) from the standard files are also shown for comparison. The phases Zn and Cu_6_Sn_5_ are indicated by red stars and blue solid cycles, respectively.

**Figure 9:**
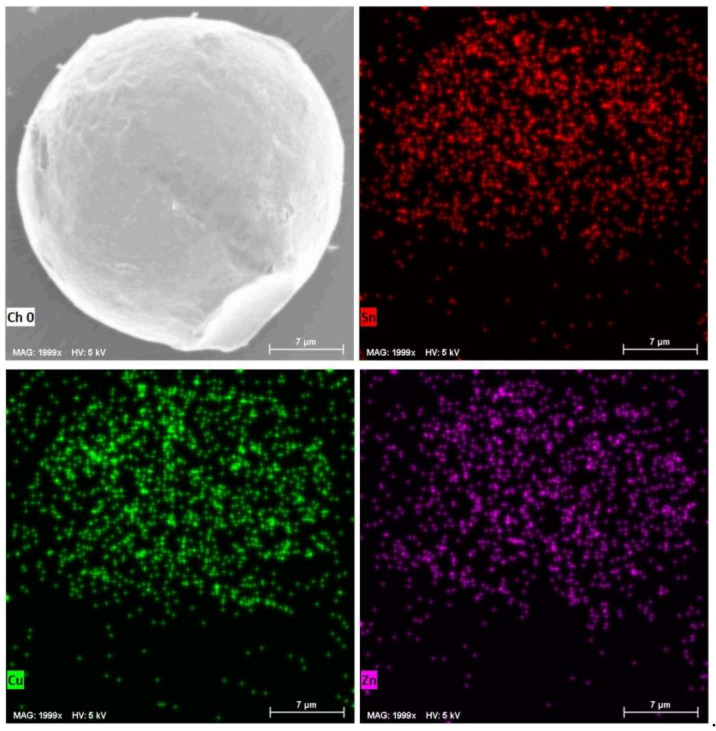
Element mapping of a Sn-0.9Cu-0.6Zn microsphere.

Figure 8 presents the XRD patterns of the Sn-0.9Cu-0.6Zn microspheres. For compassion, the diffraction data of Zn phase (JCPDS 04-0831) and Cu_6_Sn_5_ phase (JCPDS 45-1488) from the standard files are also shown. Obviously, besides the diffraction peaks of Sn, new peaks which can be attributed to the Zn phase (indicated by red stars) and the intermetallic phase Cu_6_Sn_5_ (indicated by blue solid cycles) appear, confirming the presence of both Cu and Zn in these microspheres. Furthermore, the EDS elemental mapping of a Sn-0.9Cu-0.6Zn microsphere also demonstrates that both Cu and Zn elements are present on the surface of the microsphere (Figure 9). These results suggest that the molten LiCl–KCl–CsCl salt-based metal emulsion method can be extended to the synthesis of Sn-based multicomponent microspheres. Since the chloride salts can be recycled by means of the evaporation of water and are stable at a high temperature, our results indicate that the LiCl–KCl–CsCl salt-based metal emulsion method might also serve as an environmentally friendly means for the synthesis of other metals and their alloy microspheres with a melting point higher than that of Sn.

### 2.2. In LiNO_3_-NaNO_3_-KNO_3_ System

**Figure 10:**
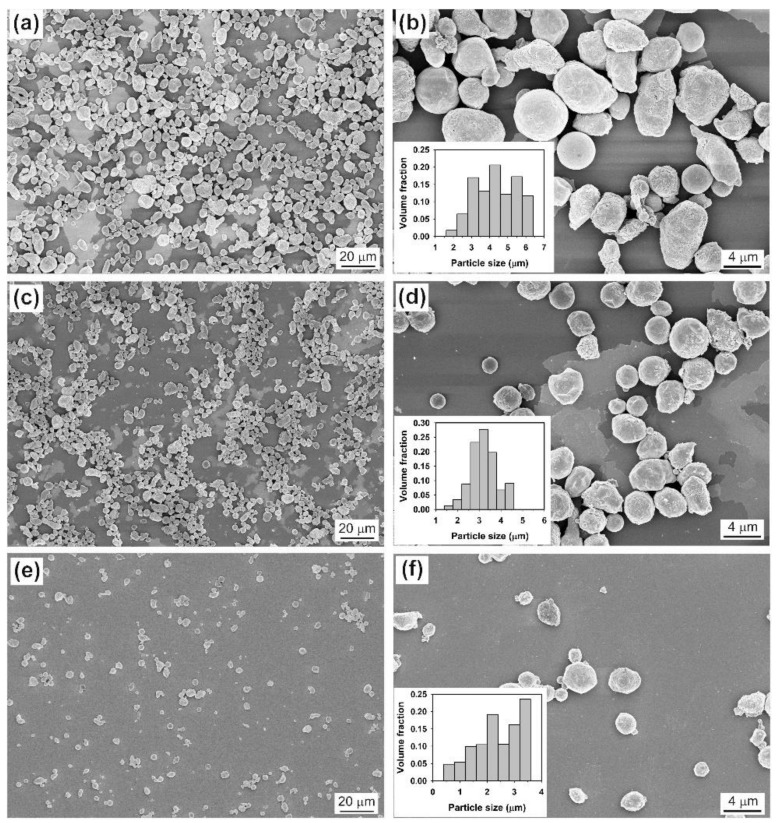
SEM images of the products obtained in LiNO_3_-NaNO_3_-KNO_3_ system under different sonication times at an ultrasonic power of 12 W: (**a**,**b**) 1 min, (**c**,**d**) 2 min, and (**e**,**f**) 5 min. Other conditions: volume ratio of salts to metal Sn = 1000:1. Inset is the corresponding volume-based particle size distribution.

In this section, we use the synthesis of Sn microspheres as an example to illustrate the effect of molten nitrate salts on the morphology and phase composition of the product. Unlike the LiCl–KCl–CsCl eutectic, the LiNO_3_-NaNO_3_-KNO_3_ eutectic has a much lower melting point (close to 120 °C) [22]. Therefore, an initial temperature of 260 °C, namely 30 °C above the melting point of Sn, is chosen to conduct the experiment. In the LiCl–KCl–CsCl system, our experimental results reveal that a low-power ultrasound is not effective for dispersing liquid Sn. In the LiNO_3_-NaNO_3_-KNO_3_ system, however, it is interesting to note that a very low ultrasonic power (e.g., 12 W, the lowest power provided by our instrument) can be used to disperse liquid Sn. Figure 10 presents the typical SEM images of the products formed by changing the sonication time at an applied power of 12 W. Similarly to the result observed in the LiCl–KCl–CsCl system with an applied power of 1080 W, the particle size of the product shows a decreasing trend as the sonication time increases from 1 to 5 min. However, a significant difference between two cases is that the particles formed in the LiNO_3_-NaNO_3_-KNO_3_ system are much smaller than those in the LiCl–KCl–CsCl system, despite the fact that the former uses a much lower ultrasonic power. For example, when the sonication time reaches 1 or 5 min, the particle size of the product obtained in the LiNO_3_-NaNO_3_-KNO_3_ system is generally less than 10 (Figure 10a–d) or 4 µm (Figure 10e,f).

**Figure 11:**
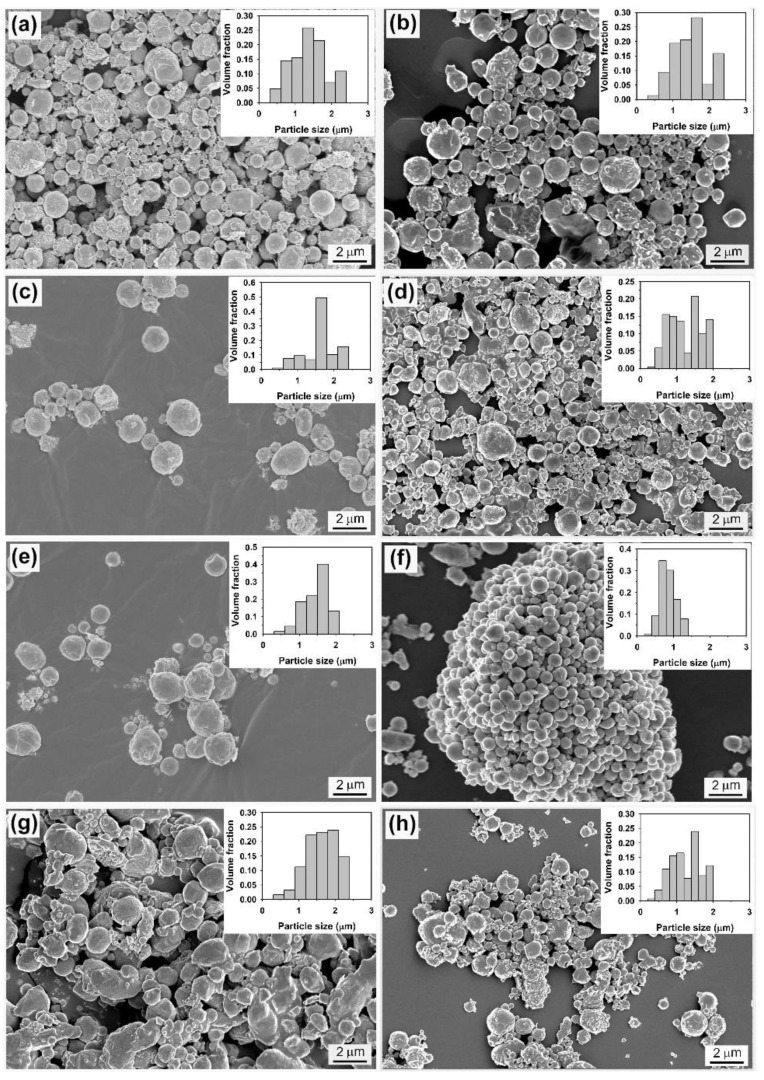
SEM images of the products obtained in LiNO_3_-NaNO_3_-KNO_3_ system under different experimental conditions: (**a**) 480 W—5 min—1000:1, (**b**) 840 W—5 min—1000:1, (**c**) 1080 W—5 min—1000:1, (**d**) 1080 W—10 min—1000:1, (**e**) 1080 W—5 min—600:1, (**f**) 1080 W—5 min—200:1, (**g**) 1080 W—5 min—50:1, and (**h**) 1080 W—5 min—25:1.

In order to further make a comparison between the results obtained in two different salt systems under similar conditions, a high ultrasonic power was also employed to disperse Sn into the molten LiNO_3_-NaNO_3_-KNO_3_ eutectic. Figure 11 presents the SEM images of the products formed under different experimental conditions. It is obvious that the change in ultrasonic power (480–1080 W), sonication time (5–10 min), or volume ratio of salts to metal (25:1–1000:1) has no significant impact on the size of the resulting materials, and the particle size of the product is generally in the range of hundreds of nanometers to 2.5 µm, which is slightly smaller than those obtained at a sonication time of 5 min under an applied power of 12 W. This suggests that the interaction between droplet breakup and droplet coalescence in the metal emulsion tends to reach an equilibrium when the size of the droplets reduces to a given value. Compared to the size of Sn particles obtained in the LiCl–KCl–CsCl system, however, the particle size of the product obtained in the LiNO_3_-NaNO_3_-KNO_3_ system is much smaller. From a thermodynamical point of view, the energy required to break up big metal droplets into small ones is proportional to the value of interfacial tension between molten nitrate salts and liquid Sn and is inversely proportional to the value of radius of metal droplets; the much lower ultrasonic power needed and the much smaller particle size of the product observed in LiNO_3_-NaNO_3_-KNO_3_ system suggest that the interfacial tension between the molten nitrate salts and liquid Sn is much lower than that between the molten chloride salts and liquid Sn.

**Figure 12:**
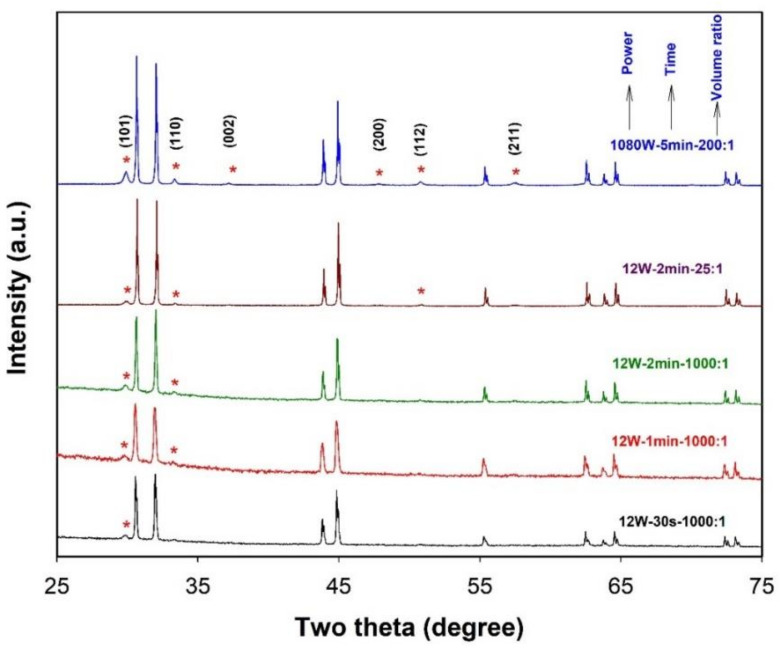
XRD patterns of the products obtained in LiNO_3_-NaNO_3_-KNO_3_ system under different experimental conditions. The diffraction peaks assigned to the SnO phase are indicated by stars.

To understand the above experimental observations, the phase composition of the products formed in the LiNO_3_-NaNO_3_-KNO_3_ eutectic under several different conditions was investigated, and the results are shown in Figure 12. In the XRD patterns of the products prepared under different conditions (whether the sonication time is short or long, the volume ratio is big or small, and the ultrasonic power is high or low), besides the diffraction peaks which can be indexed to metal Sn with a tetragonal structure (space group I41/amd, JCPDS 04-0673), new peaks (indicated by stars) which can be attributed to the phase of SnO with a tetragonal structure (space group P4/nmm, JCPDS 06-0395) appear. This hints that the oxidation of Sn droplets occurs during the dispersion of Sn into the LiNO_3_-NaNO_3_-KNO_3_ eutectic by acoustic cavitation. It has been reported that the LiNO_3_-NaNO_3_-KNO_3_ ternary system shows a very good long-term thermal stability at a temperature of 425 °C [22]. Since the measured temperature under an ultrasonic power of 12 W is well below 425 °C, the formation of SnO is believed to be a result of ultrasound-induced decomposition of nitrates where the in situ generated O_2_ can react with the metal droplets at the interface between the metal and salts. We speculate that the decomposition of nitrates might be related to the instantaneous local high temperature caused by acoustic cavitation.

Our above results reveal that there exists an ultrasound-induced interfacial oxidation reaction in the LiNO_3_-NaNO_3_-KNO_3_ system. The occurrence of such an interfacial reaction is expected to affect the size of metal droplets in the emulsion in two ways. First, it is reported that the occurrence of an interfacial reaction can significantly reduce the interfacial tension between two liquid phases in the emulsion [17,18,21]. The interfacial tension between liquid metal and molten salts ($\gamma_{metal/salts}$) can be expressed according to the expression derived by Girifalco and Good [25].

(1) $$\gamma_{metal/salts}=\gamma_{metal}+\gamma_{salts}-2\phi {\left(\gamma_{metal}\cdot \gamma_{salts}\right)}^{\frac{1}{2}}$$

where $\gamma_{metal}$ and $\gamma_{salts}$ are, respectively, the surface tensions of liquid metal and molten salts, and *ϕ* is the interaction parameter between two phases and its value increases as the interaction increases. Ye and Sahai reported that that the occurrence of an interfacial reaction could greatly increase the value of *ϕ* and thus reduce the interfacial tension between liquid Al and molten salts [21]. Therefore, in our case, the reaction between Sn liquid droplets and O_2_ in situ generated by nitrate decomposition is also expected to increase the interaction between Sn and salts and thus reduce their interfacial tension. As a result, smaller Sn droplets can be formed in the emulsion. Second, the occurrence of such an interfacial oxidation reaction leads to the formation of SnO with a melting point that is much higher than the experimental temperature. This indicates that the solid phase SnO might serve as the stabilizer of the emulsion, as in the case of Pickering emulsions, where solid particle-stabilized droplets are suspended in an immiscible continuous liquid phase [26].

### 2.3. Phase Change Properties of Sn-Based Microspheres

**Figure 13:**
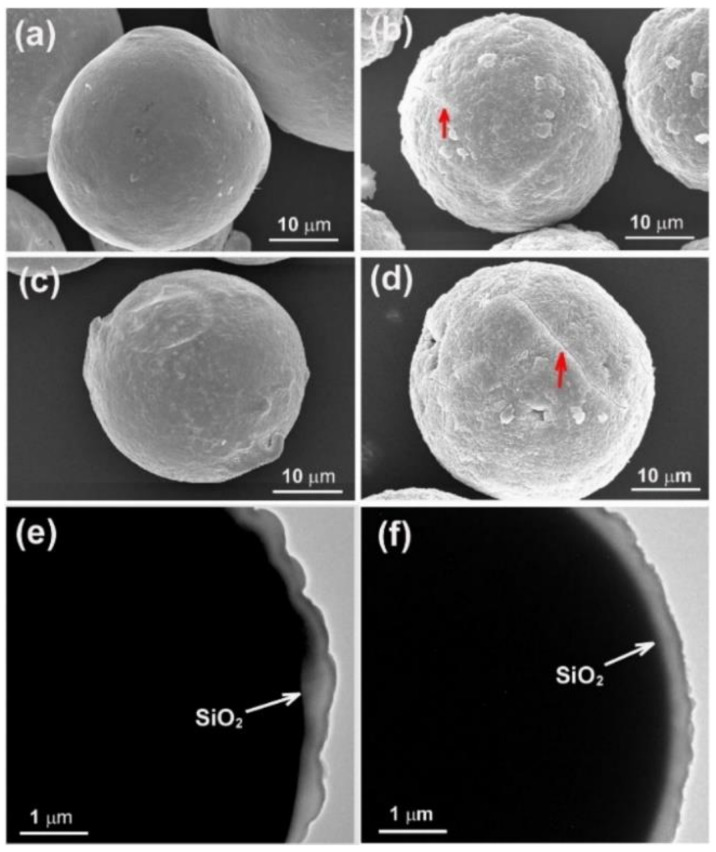
SEM images of Sn-0.6Cu (**a**,**b**) and Sn-0.9Cu-0.6Zn (**c**,**d**) microspheres before (**a**,**c**) and after (**b**,**d**) encapsulated in SiO_2_. TEM images of enlarged part of a Sn-0.6Cu (**e**) or Sn-0.9Cu-0.6Zn (**f**) microsphere. Cracks are indicated by red arrow and the white arrows point to the SiO_2_ layer.

Owing to having a melting point close to some nitrate eutectic salt-based PCMs [3], encapsulated Sn microspheres may serve as substitutes for nitrate-based PCMs in thermal energy storage. However, an extremely high undercooling is normally observed for encapsulated Sn microparticle spheres, and thus greatly restricts their practical application as PCMs [27,28]. For SiO_2_-encapsulated Sn microspheres of about 40 µm in diameter, for instance, an undercooling of about 76 °C can be found in the Bao’s work [8]. A value of about 78 °C can also be observed for Al_2_O_3_-microencapsulated Sn particles [10]. Although no work on the undercooling suppression of microencapsulated Sn-based PCMs can be found in the literature, it has been reported that doping traces of elements can reduce the undercooling of bulk Sn [29,30]. For example, the introduction of Cu (0.2–0.9 wt.%) [29,30], especially both Cu (0.9 wt.%) and Zn (0.6 wt.%) [30], into Sn is found to be an effective way to depress the undercooling of Sn-based solders. Therefore, to make a comparison with Sn-based bulk solders and to understand the element doping effect on phase change properties, especially the undercooling, of Sn microspheres, the as-prepared Sn-Cu and Sn-Cu-Zn microspheres with a similar amount of Cu or Zn were encapsulated in SiO_2_ and then studied by means of DSC. Figure 13a–d present the typical SEM images of Sn-0.6Cu and Sn-0.9Cu-0.6Zn microspheres before and after being encapsulated in SiO_2_. In general, the surfaces of metal microspheres before encapsulation are smoother than those after encapsulation. In addition, cracks (indicated by red arrows in Figure 13), a result of the removal of water in the SiO_2_ layer during the drying process, are often observed on the surface of encapsulated microspheres. The TEM images, shown in Figure 13e,f, reveal that the thickness of the SiO_2_ layer is about 300 nm.

**Figure 14:**
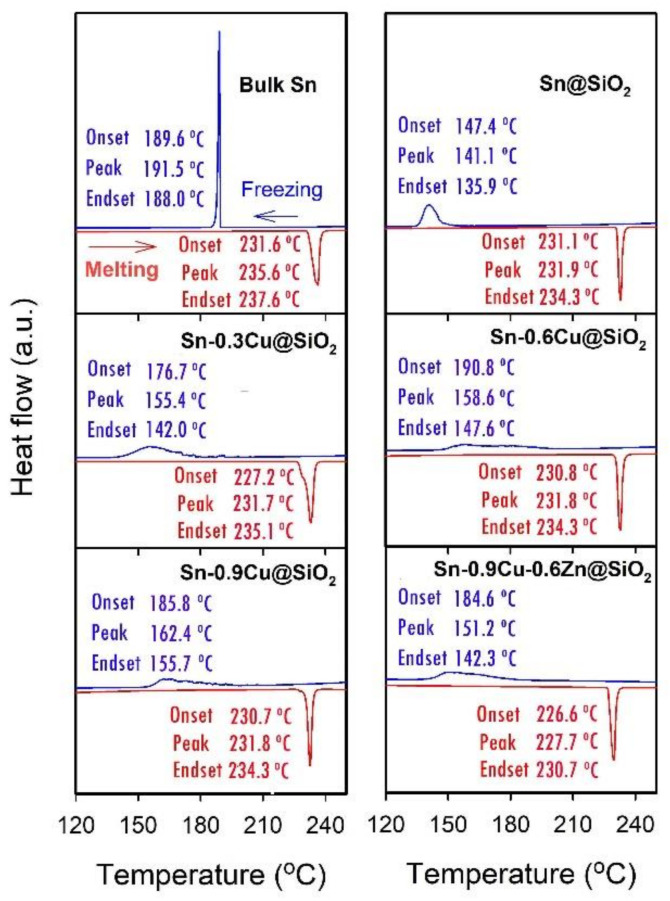
DSC curves of SiO_2_-encapsulated Sn-Cu and Sn-Cu-Zn microspheres of different compositions. The results obtained from bulk Sn and Sn@SiO_2_ microspheres are also shown for comparison.

**Figure 15:**
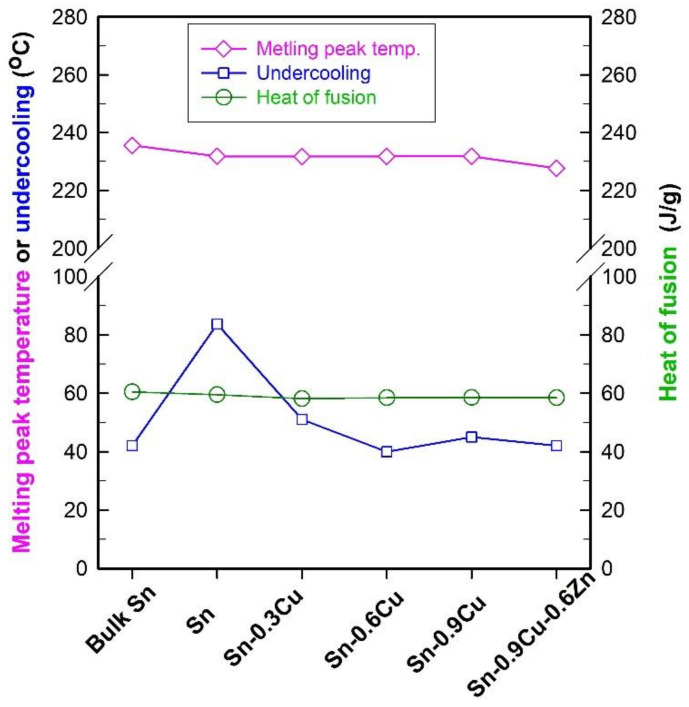
Phase change properties of SiO_2_-encapsulated Sn-Cu and Sn-Cu-Zn microspheres of different compositions. The results obtained from bulk Sn and Sn@SiO_2_ microspheres are also shown for comparison.

In order to make a comparison with the results regarding Sn-based bulk solders reported in the literature [29,30], the undercooling in this work is also defined as the difference between the onset temperatures of melting and solidification measured by DSC. Figure 14 presents the DSC curves of encapsulated Sn-Cu and Sn-Cu-Zn microspheres of different compositions obtained at a ramp rate of 10 °C/min, and the obtained phase change properties of these microspheres, including undercooling, melting peak temperature, and heat of fusion, are shown in Figure 15. For the sake of comparison, the results obtained from bulk Sn and encapsulated Sn microspheres are also shown. From Figure 14 and Figure 15, it is clear that doping with a small amount of these elements has almost no impact on the heat of fusion, and except for a slight decrease observed in Sn-0.9Cu-0.6Zn microspheres, the melting peak temperature is also not changed obviously after doping with a trace of Cu. However, the undercooling of Sn is notably reduced after doping with Cu or both Cu and Zn. For example, the undercoolings of encapsulated Sn-0.3Cu, Sn-0.6Cu, Sn-0.9Cu and Sn-0.9Cu-0.6Zn microspheres are, respectively, about 51, 40, 45 and 42 °C, which are much lower than the value of about 84 °C observed in encapsulated Sn microspheres. In addition, a size effect on the undercooling is observed [31]. The undercooling of bulk Sn is about 42 °C, but the encapsulated Sn microspheres have an undercooling of 84 °C. This size effect also exists in the Sn-Cu and Sn-Cu-Zn microspheres. Compared with the undercooling of 28.9 °C for Sn-0.9Cu and 6.8 °C for Sn-0.9Cu-0.6Zn solders reported in the literature [30], the encapsulated Sn-0.9Cu microspheres, especially encapsulated Sn-0.9Cu-0.6Zn microspheres, have a much higher undercooling level despite the presence of a new phase, Cu_6_Sn_5_, which can serve as a nucleation site to induce heterogeneous nucleation. In addition, a very broad freezing peak is often observed in these doped Sn microspheres, indicating a broad freezing range and thus a low temperature needed for them to totally release latent heat. These results indicate that doping with 0.9 wt.% of Cu and 0.6 wt.% of Zn is less efficient for suppressing the undercooling of Sn microspheres than that of bulk Sn solder. Therefore, the preparation of Cu- and Zn-doped Sn microspheres with low undercooling and a narrow freezing range remains a challenge, and work is underway in our group to achieve such a goal.

## 3. Materials and Methods

### 3.1. Materials and Chemicals

All chemicals were of analytical grade and were used as received. Except for Sn (≥99.5%, MCLean Biochemical Technology Co., Ltd., Shanghai, China) and ethanol (purchased from Anhui Ante Food Co., Ltd., Suzhou, China), all other reagents, including Zn particles (≥99.9%) and Cu powder (≥99.9%), were obtained from Aladdin Chemical Reagent Co., Ltd., Shanghai, China.

### 3.2. Preparation of Sn-Based Materials via a Molten Salt-Based Metal Emulsion Method and Encapsulation of Sn-Based Microspheres

For the preparation of Sn microspheres in two different types of molten salts. A given amount of bulk Sn particles was firstly added into a quartz tube containing a certain amount of molten LiCl–KCl–CsCl eutectic at 300 °C or LiNO_3_-NaNO_3_-KNO_3_ eutectic at 260 °C under argon gas protection, and then the mixture was emulsified via ultrasonic cavitation at a certain power (20 KHz, 3 s interval between every 2 s duty) for a given time with the distance between the sonotrode tip (φ20 mm) and the molten salt–metal interface being about 8 mm. Then, the metal emulsion was quickly cooled to room temperature by dumping them into an Ar-filled steel container which was put in an ice-water bath. The chloride salts were separated from the metals by dissolving them in deionized water. After the removal of the salts, the solid product was washed with deionized water and then dried.

Preparation of Sn-Cu or Sn-Cu-Zn microspheres in chloride salts. Typically, about 3.0 g of Sn and a given amount of Cu powder were put into a corundum tube which was filled with Ar gas. Then, the tube was heated to 1000 °C and kept at that temperature for 1 h (for preparation of Sn-Cu-Zn, the liquid metals were cooled down to 600 °C, and a given amount of Zn powder was added under Ar gas protection and then kept at 600 °C for 1 h under stirring). After that, the liquid metals were rapidly cooled down by dumping them into an Ar-filled steel container which was put in an ice-water bath. The above cooled mixture was transferred into a quartz tube, covered by about 22.1 g of LiCl–KCl–CsCl eutectic (54.4 wt.% CsCl, 30.3 wt.% LiCl, and 15.3 wt.% KCl), and then heated to 600 °C under argon gas protection. After being kept at 600 °C for about 10 min, the mixture was cooled to 380 °C and then emulsified via ultrasonic cavitation (20 KHz, 960 W, 3 s interval between every 2 s duty) for about 5 min. Then, the resulting emulsion was quickly cooled and the salts were removed by the same procedure as used in the preparation of Sn microspheres to obtain Sn-Cu or Sn-Cu-Zn microspheres.

Encapsulating Sn-based microspheres in SiO_2_. Typically, about 0.5 g of metal microparticles was added into a plastic container. Then, 0.6 mL of freshly prepared NH_4_F aqueous solution (containing 0.001gNH_4_F), 3 mL of ethanol, and about 0.01 mL of Span-80 were successively added into the container. After Span-80 was dissolved under stirring, 10 mL of cyclohexane was added, and then the container was placed into a water bath. The Sn-containing mixture was dispersed into cyclohexane by probe sonication (sonotrode tip diameter = 10 mm, 240 W). After 2 min of sonication, about 2.3 mL of tetraethyl orthosilicate (TEOS) was added dropwise into the tube in 2 min under ultrasonic cavitation. After ultrasonic cavitation for another 20 min, the solid deposit in the bottom of the container was collected by centrifugation (1000 rpm), washed with water and ethanol, and finally dried at 65 °C in vacuum.

### 3.3. Characterization

The morphology of the samples was examined using a scanning electron microscope (SEM, Hitachi S-4700) operating at 15 kV. The concentration of elements was measured by an energy-dispersive X-ray spectrometer (EDS) attached to SEM. The phase composition was analyzed by X-ray diffraction (XRD), which was performed on a Thermo ARL XTRA X-ray diffractometer (Thermo Fisher Scientific) using a Cu K α X-ray source. The microstructure investigations were performed with a Tecnai G2 F30 S-Twin transmission electron microscopy operating at 300 kV. Measurements of solid liquid phase change temperatures and latent heat capacities of PCMs were carried out by using a differential scanning calorimeter instrument (DSC, Mettler Toledo) under nitrogen atmosphere and at a heating/cooling rate of 10 °C/min. The latent heat was calculated through numerical integration of the peak using the inherent software of the DSC instrument.

## 4. Conclusions

In summary, a comparative study of the metal emulsion-based synthesis of Sn-based materials in two types of molten salts has been presented and the properties of Sn, Sn-Cu and Sn-Cu-Zn microsphere phase change materials prepared in chloride salts have been evaluated by DSC. In the LiCl–KCl–CsCl system, we can prepare well-shaped Sn microspheres with controlled sizes, e.g., 1–15 µm or several micrometers to about 30 µm in diameter, by adjusting the preparation parameters. In the LiNO_3_-NaNO_3_-KNO_3_ system, however, an oxidation reaction between Sn liquid droplets and O_2_ in situ generated by ultrasound-induced decomposition of nitrates is observed. Such an interfacial reaction favors the formation of small Sn liquid droplets by lowering the Sn–salt interfacial tension and hindering the coalescence of Sn droplets due to the formation of a solid SnO phase. As a result, a very low ultrasonic power can be used to disperse liquid Sn into nitrates, and Sn-based particles typically in the diameter range of hundreds of nanometers to several micrometers can be produced. A DSC study of Sn-based microspheres encapsulated in SiO_2_ reveals that Cu (0.3–0.9 wt.%) or Cu-Zn (0.9 wt.% Cu and 0.6% Zn) doping can reduce the undercooling of Sn via raising the onset freezing temperature, but a broad freezing peak observed in these doped microspheres, along with a still much higher undercooling compared to those of reported Sn-Cu or Sn-Cu-Zn solders, suggests the existence of a size effect and that a low temperature is still needed for these microspheres to totally release latent heat.

## Sample Availability

Samples of the compounds are available from the authors.
